# Reported Adverse Health Effects in Children from Ingestion of Alcohol-Based Hand Sanitizers — United States, 2011–2014

**DOI:** 10.15585/mmwr.mm6608a5

**Published:** 2017-03-03

**Authors:** Cynthia Santos, Stephanie Kieszak, Alice Wang, Royal Law, Joshua Schier, Amy Wolkin

**Affiliations:** ^1^Division of Environmental Hazards and Health Effects, National Center for Environmental Health, CDC; ^2^Emory University School of Medicine, Atlanta, Georgia; ^3^Office of Public Health Preparedness and Response, CDC.

Hand sanitizers are effective and inexpensive products that can reduce microorganisms on the skin, but ingestion or improper use can be associated with health risks. Many hand sanitizers contain up to 60%–95% ethanol or isopropyl alcohol by volume, and are often combined with scents that might be appealing to young children. Recent reports have identified serious consequences, including apnea, acidosis, and coma in young children who swallowed alcohol-based (alcohol) hand sanitizer (*1*–*3*). Poison control centers collect data on intentional and unintentional exposures to hand sanitizer solutions resulting from various routes of exposure, including ingestion, inhalation, and dermal and ocular exposures. To characterize exposures of children aged ≤12 years to alcohol hand sanitizers, CDC analyzed data reported to the National Poison Data System (NPDS).* The major route of exposure to both alcohol and nonalcohol-based (nonalcohol) hand sanitizers was ingestion. The majority of intentional exposures to alcohol hand sanitizers occurred in children aged 6–12 years. Alcohol hand sanitizer exposures were associated with worse outcomes than were nonalcohol hand sanitizer exposures. Caregivers and health care providers should be aware of the potential dangers associated with hand sanitizer ingestion. Children using alcohol hand sanitizers should be supervised and these products should be kept out of reach from children when not in use.

In 2005, the annual rate of intentional alcohol hand sanitizer exposure was 0.68 per 1 million U.S. residents (95% confidence interval [CI] = 0.17–1.20) (*4*). During 2005–2009, this rate increased, on average, by 0.32 per 1 million per year (95% CI = 0.11–0.53; p = 0.02) (*4*). Young children, including infants, are more likely to develop complications from alcohol intoxication than are older children and teens. Younger children have decreased liver glycogen stores, which increase their risk of developing hypoglycemia, and have various pharmokinetic factors, which make them more susceptible to developing toxicity from alcohol (*5*–*9*). To characterize pediatric alcohol hand sanitizer exposures in the United States, data reported by poison centers in all states to NPDS among children aged ≤12 years during January 1, 2011–December 31, 2014 were analyzed. Analyses were stratified by age group (0–5 years and 6–12 years). Hand sanitizer exposures were defined as a poison center call reporting an exposure to either ethanol-based or isopropanol-based sanitizer solutions (alcohol hand sanitizer exposure) or a nonalcohol sanitizer product (nonalcohol hand sanitizer exposure). Calls reporting co-exposures to other agents were excluded to minimize confounding effects.

Descriptive statistics were compiled for exposed children’s age, year and season of exposure, intentionality of exposure, route of exposure (ingestion, inhalation, dermal, or ocular), reported health effects (e.g., drowsiness, eye irritation, nausea, vomiting, etc.), and outcome,^†^ and were compared for alcohol and nonalcohol hand sanitizers and age group. An exposure was coded by poison centers as unintentional if it was considered to be accidental or inadvertent. Deliberate exposures, because of deliberate misuse or abuse for example, were considered intentional. An exposure was considered to have resulted in an adverse health effect if at least one symptom (e.g., abdominal pain, nausea, vomiting, etc.) was reported. Categorical data comparisons were performed using the chi-square test or, when cell sizes were <5, Fisher’s exact test. Significance was defined as p<0.05. Statistical software was used for the analysis.

During 2011–2014, a total of 70,669 hand sanitizer exposures in children aged ≤12 years were reported to NPDS, including 65,293 (92%) alcohol exposures, and 5,376 (8%) nonalcohol exposures (Table 1). The number and percentage of each type of reported exposure was similar during each of the 4 years. Overall, 64,488 (91%) exposures occurred in children aged ≤5 years, and 6,181 (9%) occurred in children aged 6–12 years. There was no association between sanitizer type and year. Among all children, ingestion accounted for approximately 95% of reported exposures, including 97% of exposures among children aged ≤5 years (97.0% alcohol and 96.3% nonalcohol exposures) and 74% among children aged 6–12 years (74.0% alcohol and 72.0% nonalcohol exposures). A higher percentage of older children (aged 6–12 years) had intentional exposures to alcohol hand sanitizers (866; 15.0%) than to nonalcohol hand sanitizers (40; 8.0%) (p<0.001). This association was not found in younger children (aged ≤5 years). Ocular exposures to hand sanitizers were more common in older children (24.8% overall, 24.4% alcohol, and 29.0% nonalcohol) than among younger children (3.0% overall, 3.0% alcohol, and 3.2% nonalcohol). Although there was no seasonal variation in reported exposure to either hand sanitizer type among younger children, exposure frequency among older children was lower for both hand sanitizer types during the summer months (Figure).

**TABLE 1:** Exposures to alcohol and nonalcohol hand sanitizer products among children aged ≤12 years reported to poison centers, by sanitizer type, year, age group, exposure route, and intentionality — United States, National Poison Data System, 2011–2014

Year	No. (%) of exposures		
	Alcohol	Nonalcohol	Total
**Total**	**65,293 (92.4)**	**5,376 (7.6)**	**70,669**
2011	15,971 (92.5)	1,286 (7.5)	17,257
2012	16,571 (92.4)	1,355 (7.6)	17,926
2013	16,423 (92.5)	1,338 (7.5)	17,761
2014	16,328 (92.1)	1,397 (7.9)	17,725
**Age group 0–5 yrs**			
**Total**	**59,612 (92.4)**	**4,876 (7.6)**	64,488 (91.2)*
**Exposure route**			
Ingestion	57,825 (97.0)	4,698 (96.3)	62,523 (97.0)
Inhalation	74 (0.1)	10 (0.2)	84 (0.1)
Dermal	2,385 (4.0)	135 (2.8)	2,520 (3.9)
Ocular	1,782 (3.0)	157 (3.2)	1,939 (3.0)
**Intentionality**			
Intentional	37 (0.1)	1 (0.0)	38 (0.1)
Unintentional	59,575 (99.9)	4,875 (100.0)	64,450 (99.9)
**Age group 6–12 yrs**			
**Total**	**5,681 (91.9)**	**500 (8.1)**	6,181 (8.7)*
**Exposure route**			
Ingestion	4,204 (74.0)	351 (70.2)	4,555 (74.0)
Inhalation	81 (1.4)	6 (1.2)	87 (1.4)
Dermal	180 (3.2)	9 (1.8)	189 (3.1)
Ocular	1,387 (24.4)	145 (29.0)	1,532 (24.8)
**Intentionality**			
Intentional	866 (15.2)	40 (8.0)	906 (14.7)
Unintentional	4,815 (84.8)	460 (92.0)	5,275 (85.3)

*Percentage of total exposures.

**FIGURE:**
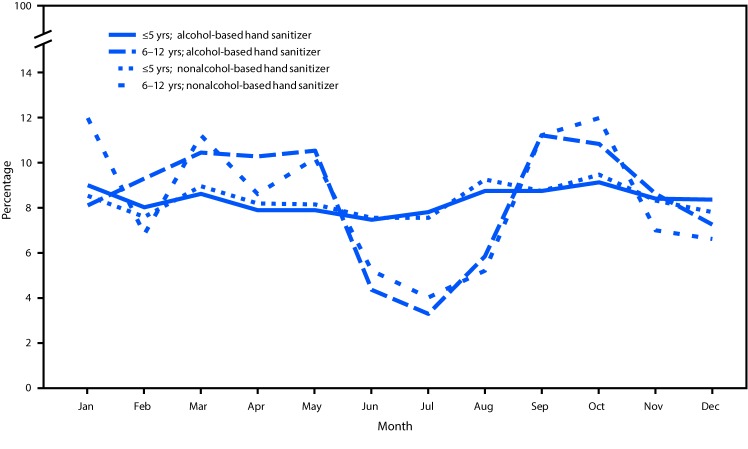
Percentage of exposures from alcohol-based and nonalcohol-based hand sanitizer products in children aged ≤5 years and aged 6–12 years reported to poison centers, by month — United States, National Poison Data System, January 1, 2011–December 31, 2014

Overall, 8,219 (12%) patients had at least one reported symptom, including 7,703 (12%) children who ingested alcohol products, and 516 (10%) who ingested nonalcohol products. Adverse health effects were more likely to be reported for alcohol hand sanitizer exposures (p<0.001). The most common adverse health effects for both hand sanitizer types were ocular irritation (2,577; 31.4%) and vomiting (1,872; 22.8%). Conjunctivitis (862; 10.5%), oral irritation (782; 9.5%), cough (705; 8.6%), and abdominal pain (323; 3.9%) were also reported (Table 2). Rare health effects included coma (five), seizures (three), hypoglycemia (two), metabolic acidosis (two), and respiratory depression (two). Those rare effects occurred more frequently among children with alcohol hand sanitizer exposures, but the differences were not statistically significant when the rare health effects were analyzed individually. Alcohol hand sanitizers were significantly associated with worse outcomes (compared with no effect outcomes) when both age groups were analyzed (p = 0.02). Approximately two thirds (66%) of children with exposures were not followed to determine outcome (Table 2). Among patients who were followed (23,828), exposure to alcohol hand sanitizers had no reported effect in 17,441 (85%) of the younger children. In contrast, 1,005 (50%) of the older children had no reported effect to alcohol hand sanitizer exposure. No deaths were reported.

**TABLE 2:** Most common adverse health effects and outcomes experienced by children with exposure to alcohol and nonalcohol hand sanitizers, by age group — United States, 2011–2014

Characteristic	No. (%)				
	Alcohol	Nonalcohol	Alcohol	Nonalcohol	Total
	<5 yrs	<5 yrs	6–12 yrs	6–12 yrs	
**Total**	**59,612**	**4,876**	**5,681**	**500**	**70,669**
**Symptoms**					
Reported symptoms	5,867 (9.8)	379 (7.8)	1,836 (32.3)	137 (27.4)	**8,219 (11.6)**
Ocular irritation	1,306 (22.3)*	97 (25.6)*	1,080 (58.8)*	94 (68.6)*	**2,577 (31.4)**
Vomiting	1,606 (27.4)*	129 (34.0)*	129 (7.0)	8 (5.8)*	**1,872 (22.8)**
Red eye/Conjunctivitis	492 (8.4)	33 (8.7)	316 (17.2)*	21 (15.3)*	**862 (10.5)**
Oral irritation	699 (11.9)*	26 (6.9)	55 (3.0)	2 (1.5)	**782 (9.5)**
Cough	651 (11.1)	43 (11.4)*	11 (0.6)	0 (0.0)	**705 (8.6)**
Abdominal pain	173 (3.0)	10 (2.6)	135 (7.4)*	5 (3.7)	**323 (3.9)**
**Outcomes**					
No effect	17,441 (29.3)	956 (19.6)	1,005 (17.7)	71 (14.2)	**19,473 (27.6)**
Minor outcome^†^	2,957 (5.0)	188 (3.9)	962 (16.9)	85 (17.0)	**4,192 (5.9)**
Moderate outcome^§^	105 (0.2)	4 (0.1)	45 (0.8)	4 (0.8)	**158 (0.2)**
Major outcome^¶^	4 (0.0)	0 (0.0)	1 (0.0)	0 (0.0)	**5 (0.0)**
Not followed	39,105 (65.6)	3,728 (76.5)	3,668 (64.6)	340 (68.0)	**46,841 (66.3)**

* The three most commonly reported symptoms per column.

^†^ The patient exhibited some symptoms as a result of the exposure, but they were minimally bothersome to the patient. The symptoms usually resolved rapidly and often involved skin or mucous membrane manifestations. The patient returned to a preexposure state of well-being and had no residual disability or disfigurement.

^§^ The patient exhibited symptoms as a result of the exposure that were more pronounced, more prolonged, or more of a systemic nature than minor symptoms. Usually some form of treatment was or would have been indicated. Symptoms were not life-threatening and the patient returned to a preexposure state of well-being with no residual disability or disfigurement.

^¶^ The patient exhibited symptoms as a result of the exposure that were life-threatening or resulted in significant residual disability or disfigurement.

## Discussion

In this analysis, alcohol hand sanitizer exposures, the majority of which were ingestions, were associated with worse outcomes than nonalcohol hand sanitizer exposures. Older children (aged 6–12 years) were more likely to report intentional ingestion and to have adverse health effects and worse outcomes than were younger children, suggesting that older children might be deliberately misusing or abusing alcohol hand sanitizers. These data also indicate that, among older children, exposures occur less frequently during the summer months. The reason for this seasonal trend is unknown but might be associated with flu season or more ready access to hand sanitizers during the school year. Some schools might require or ask children to purchase and carry hand sanitizers, which might contribute to the higher number of exposures during the school year. A study examining Texas poison center data from 2000 to 2013 found that, among 385 adolescents who ingested hand sanitizer, 35% of ingestions occurred at school (*10*).

The findings in this report are subject to at least three limitations, which might have led to an underestimate of the total number of alcohol and nonalcohol hand sanitizer exposures. First, calls involving hand sanitizer exposures and another exposure were excluded. Second, the codes indicating an alcohol hand sanitizer exposure also were changed in 2010 and might have been initially underused. Finally, public and health care providers, including emergency department providers, also might not have reported all alcohol or nonalcohol hand sanitizer exposures to poison centers. Moreover, poison center data are also subject to inherent biases such as selection bias (e.g., if poisoning is unrecognized as a cause) or information bias (e.g., recall or interviewer bias). An important example of information bias in this study could be exposure intentionality being incorrectly coded because of inaccurate or subjective history obtained by the caller. 

Hand washing with soap and water is the recommended method of hand hygiene in non–health care settings. If soap and water are not available, use of a hand sanitizer that contains at least 60% alcohol is suggested.^§^ Other options, such as nonalcohol hand sanitizers or wipes, can be used if soap and water or alcohol hand sanitizers are not available or practical. In September 2016, the Food and Drug Administration issued a rule banning the use of triclosan, triclocarban, and 17 other chemicals in consumer hand and body antibacterial soaps and washes because of health and bacterial resistance concerns. However, this ban does not apply to hand sanitizers, hand wipes, or antibacterial soaps used in a health care setting.^¶^ Hand washing with plain soap and water is safe and effective and does not carry these associated risks.

Increasing awareness of the potential dangers associated with intentional or unintentional ingestion of alcohol hand sanitizers might help encourage proper use and avoid adverse outcomes. Using alcohol hand sanitizers correctly, under adult supervision, and with proper child safety precautions and making sure they are stored out of reach of young children might reduce unintended adverse consequences. Clinicians evaluating pediatric patients with clinical signs and symptoms consistent with alcohol toxicity, such as nausea, vomiting, respiratory depression, and drowsiness or laboratory results consistent with ethanol or isopropanol toxicity, should consider the possibility of an alcohol hand sanitizer ingestion and contact their local poison control center.

**Summary**

**What is already known about this topic?**

Nonrecommended use of alcohol-based (alcohol) hand sanitizers, including intentional or unintentional ingestion, might be associated with greater health risks in young children than similar use of nonalcohol-based (nonalcohol) hand sanitizers.

**What is added by this report?**

During 2011–2014, 70,669 exposures to alcohol and nonalcohol hand sanitizers were reported in children aged ≤12 years to the National Poison Data System. Approximately 90% of these exposures occurred among children aged 0–5 years. Among that age group, 97% of exposures were oral ingestions. Children aged 6–12 years had more intentional exposures of alcohol hand sanitizers, suggesting this might be a potential product of abuse among older children. Older children also reported more symptoms and had worse outcomes than did younger children. Major (life-threatening) outcomes were rare. Seasonal trends in data might correlate with increased use during the school year or flu season.

**What are the implications for public health practice?**

Caregivers and health care providers need to be aware of the potential risks and dangers associated with improper use of hand sanitizer products among children and the need to use proper safety precautions to protect children. Increased parental or teacher supervision might be needed while using alcohol hand sanitizer products, especially for older children who might be abusing these products during the school year.

## Footnotes

* https://www.npds.us.

† *Minor outcomes* were defined as the occurrence of some symptoms as a result of the exposure, which were minimally bothersome to the patient, usually resolved rapidly, and often involved skin or mucous membrane manifestations, and after which, the patient returned to a preexposure state of well-being with no residual disability or disfigurement. *Moderate outcomes* were defined as the occurrence of symptoms as a result of the exposure that were more pronounced, more prolonged, or of a more systemic nature than minor symptoms; for which some form of treatment usually was or would have been indicated; were not life-threatening; and after which, the patient returned to a preexposure state of well-being with no residual disability or disfigurement. *Major outcomes* were defined as the occurrence of symptoms as a result of the exposure that were life-threatening or resulted in significant residual disability or disfigurement.

§ https://www.cdc.gov/handwashing/show-me-the-science-hand-sanitizer.html.

¶ https://www.fda.gov/ForConsumers/ConsumerUpdates/ucm378393.htm.
